# Systematic Evaluation of the Teaching Qualities of Obstetrics and Gynecology Faculty: Reliability and Validity of the SETQ Tools

**DOI:** 10.1371/journal.pone.0019142

**Published:** 2011-05-03

**Authors:** Renée van der Leeuw, Kiki Lombarts, Maas Jan Heineman, Onyebuchi Arah

**Affiliations:** 1 Department of Quality and Process Innovation, Academic Medical Center, University of Amsterdam, Amsterdam, The Netherlands; 2 Department of Obstetrics and Gynecology, Academic Medical Center, University of Amsterdam, Amsterdam, The Netherlands; 3 Department of Epidemiology, School of Public Health, University of California Los Angeles, Los Angeles, California, United States of America; 4 Center for Health Policy Research, University of California Los Angeles, Los Angeles, California, United States of America; University of Modena and Reggio Emilia, Italy

## Abstract

**Background:**

The importance of effective clinical teaching for the quality of future patient care is globally understood. Due to recent changes in graduate medical education, new tools are needed to provide faculty with reliable and individualized feedback on their teaching qualities. This study validates two instruments underlying the System for Evaluation of Teaching Qualities (SETQ) aimed at measuring and improving the teaching qualities of obstetrics and gynecology faculty.

**Methods and Findings:**

This cross-sectional multi-center questionnaire study was set in seven general teaching hospitals and two academic medical centers in the Netherlands. Seventy-seven residents and 114 faculty were invited to complete the SETQ instruments in the duration of one month from September 2008 to September 2009. To assess reliability and validity of the instruments, we used exploratory factor analysis, inter-item correlation, reliability coefficient alpha and inter-scale correlations. We also compared composite scales from factor analysis to global ratings. Finally, the number of residents' evaluations needed per faculty for reliable assessments was calculated. A total of 613 evaluations were completed by 66 residents (85.7% response rate). 99 faculty (86.8% response rate) participated in self-evaluation. Factor analysis yielded five scales with high reliability (Cronbach's alpha for residents' and faculty): learning climate (0.86 and 0.75), professional attitude (0.89 and 0.81), communication of learning goals (0.89 and 0.82), evaluation of residents (0.87 and 0.79) and feedback (0.87 and 0.86). Item-total, inter-scale and scale-global rating correlation coefficients were significant (P<0.01). Four to six residents' evaluations are needed per faculty (reliability coefficient 0.60–0.80).

**Conclusions:**

Both SETQ instruments were found reliable and valid for evaluating teaching qualities of obstetrics and gynecology faculty. Future research should examine improvement of teaching qualities when using SETQ.

## Introduction

Even experienced doctors can find it difficult to teach [1]. The importance of effective clinical teaching for the quality of future patient care is globally understood. However, formal teaching preparation is only recently being developed [2], [3]. Different features of effective faculty development - including feedback, peer mentoring and diverse educational methods within single interventions - are used to improve teaching performance [4]–[6]. Given recent duty hour reform, modernization of graduate medical education and implementation of competency based learning in residency; new tools for improvement and feedback using residents' assessments are needed [7]–[9]. Feedback appears to be a powerful tool to improve individual professional performance and leads to better clinical teaching [4], [10], [11]. Various tools have been developed to provide feedback for clinical teachers [12]–[15]. However, to our knowledge no validated and reliable tools are available to provide obstetrics and gynecology faculty with specialty-specific feedback. Although generic measurement instruments have obvious advantages for policymaking and scientific research – given their broader use and benchmark opportunities – the primary goal of a formative performance measurement system should be to provide feasible, valid and reliable feedback for faculty to use in their improvement aspirations. Therefore, measurement instruments should closely adhere to specialties' specific characteristics in line with requirements of scientific robustness. The System for Evaluation of Teaching Qualities or SETQ was developed to help fill the gap in the availability of methods to measure and improve teaching performance via feedback [16], [17]. SETQ is an integrated system designed to facilitate evaluation and improvement of individual teaching qualities of faculty of all specialties [16]–[18]. The SETQ system consists of the measurement, feedback and reflection of teaching qualities of faculty. As part of the validation of the SETQ system, this study focuses on the validation of two – a resident-completed and a faculty self-completed – measurement instruments used to generate feedback on teaching qualities for individual obstetrics and gynecology faculty. Measurement instruments need to be validated and updated for their continuous use in various local, cultural and educational contexts [19]. We are therefore exploring the psychometric qualities (reliability and validity) of the SETQ tools per specialty and in different teaching settings [16], [20]. More specifically, this article reports the initial psychometric properties of the obstetrics and gynecology SETQ instruments and it presents estimates of the number of residents' evaluations needed per faculty to generate reliable assessments.

## Methods

### The SETQ system

The SETQ system involves three phases, namely data collection, individual feedback reporting and follow-up on the results. First, data are collected by means of two secured web-based instruments, one for residents' evaluation of faculty and another for faculty's self-evaluation. Second, personal feedback reports are generated from the data and sent to individual faculty by email. Third, faculty may discuss the results with their peers or head of department. This offers an opportunity to discuss feedback and subsequently develop potential strategies for improvement.

The SETQ system started successfully in the department of anesthesiology [16]. In less than two years, other academic or teaching hospitals have adopted the SETQ system resulting in approximately 900 residents and 1050 faculty of circa 70 residency programs in 20 hospitals now participating in systematic evaluation of teaching qualities of individual faculty. It is now the most widely used system for faculty feedback in the Netherlands.

### The SETQ instruments

The two instruments underlying the SETQ system are based on the 26-item Stanford Faculty Development Program (SFDP26) instrument [12], [21], [22]. We described the development process in detail elsewhere [16], [18]. First, SETQ was implemented successfully in anesthesiology [16]. Subsequently, obstetrics and gynecology - among other residency programs - went through a similar process to develop specialty-specific instruments. The residents' and faculty's SETQ instruments each consisted of 26 core items. Each core item could be rated on a 5-point Likert-type scale: strongly disagree ‘1’, disagree ‘2’, neutral ‘3’, agree ‘4’, strongly agree ‘5’ and an additional ‘I cannot judge this’ option. Both instruments also contained two global ratings, namely ‘this faculty member is a specialist role model’ and ‘overall teaching qualities of this faculty’. For the global rating ‘overall teaching quality of this faculty’ possible responses were poor ‘1’, fair ‘2’, average ‘3’, good ‘4’ and excellent ‘5’. At the end of the questionnaire, residents were encouraged to formulate narrative feedback on strong teaching qualities as well as suggestions for improvement. We also collected data on residents' year of training and sex and faculty's age, sex, years in practice, year of first registration as an obstetrician and gynecologist and previous training in clinical teaching.

### Study Population and Setting

Seventy-seven residents and 114 faculty members of nine obstetrics and gynecology residency training programs were invited to participate in the SETQ study. In the Netherlands, residency training is organized within regional consortia of teaching hospitals, with a designated academic medical center coordinating each consortium. Faculty and residents of an academic hospital and a consortium participated.

One of the researchers (KL) introduced SETQ during regional and local meetings. Invitations to all faculty and residents were sent individually via electronic mail. The invitation emphasized the formative purpose and anonymous use of the evaluations. Residents chose whom and how many faculty to evaluate, based on whom they (had) work(ed) with the most. Each faculty could only self-evaluate. The two evaluation instruments were made electronically accessible via a dedicated SETQ web portal protected by an individual password login. Automatic email reminders were sent after 10 days, 20 days and the day before closing the data collection period.

Faculty and residents were further encouraged to participate by the head of the department in clinical meetings and by interim response updates. Data collection lasted one month for each residency program [16], [18]. Data were collected from September 2008 until September 2009. Participating clinics gave exclusive permission to use the collected data for performance and research analysis.

### Analytical Strategies

First, we described the study participants using appropriate descriptive statistics.

Second, to investigate the psychometric properties - that is whether the instruments were reliable and valid - we used five standard techniques: exploratory factor analysis, reliability coefficient calculations, item-total correlation, inter-scale correlation and scale versus global rating analysis [16], [23], [24]. To explore the teaching concepts underlying the instruments, factor analysis was conducted using the principal components technique with varimax rotation. Individual items were assigned to the composite scale on which it had the highest factor loading. For the reliability analysis, the factor structure thus found was used when calculating Cronbach's alpha as traditional measure of reliability. A Cronbach's alpha of at least 0.70 was taken as an indication of satisfactory reliability of each composite scale [25]. To check homogeneity of each composite scale, item-total correlations corrected for overlap were calculated [23]. We consider an item-total correlation coefficient of <0.3 as evidence that the item is not measuring the same construct measured by other composite scale items. We assessed the degree of overlap between the scales by estimating inter-scale correlations using Pearson's correlation coefficient. An inter-scale correlation of less than 0.70 was taken as satisfactory indication of non-redundancy of each scale [24], [26]. Subsequently, we estimated correlations between the composite scales and the two global ratings (i) faculty seen as an obstetric and gynecologic specialist role model and (ii) faculty's overall teaching qualities. Correlating each scale with each global rating provides further psychometric evidence in the validation exercise. If the SETQ instruments provided valid measures of faculty's teaching qualities, then moderate correlations with coefficients ranging from 0.40 to 0.80 should be expected between each scale and global rating. Theory and previous work suggest that each scale should correlate moderately with the global rating for being a role model, and correlate moderately or highly with the global rating for overall teaching qualities [16]–[18], [27]. The latter should be expected given that ‘teaching qualities’ is the common underlying construct in the SETQ.

Third, we calculated the number of residents' evaluations needed per faculty member for reliable assessment using previously reported psychometric methods [17], [18], [28]. As a sensitivity check, it was noted that, everything else being equal, if any new target reliability level were to be less than or equal to that observed in our study, then the required number of residents' evaluations per faculty should parallel that observed in our study. To check this assumption using our data, we re-estimated the reliability coefficients for the different sample sizes predicted by the standard methods [17], [18], [28].

All analyses were performed using PASW Statistics 18.0.0 for Mac (IBM SPSS Inc, 2009) and Microsoft Excel 2008 for Mac version 12.2.4 (Microsoft Corporation, 2007). Under Dutch law (WMO), institutional review board approval was not required for this study [29].

## Results

### Study Participants

This study included 66 residents and 99 obstetrics and gynecology faculty, representing response rates of 85.7% and 86.8% respectively. These responses yielded 613 residents' evaluations and 99 self-evaluations. Residents completed 9.3 evaluations on average, resulting in a mean of 5.3 residents' evaluations per faculty. Two-thirds (66.2%) of residents and half (50.5%) of faculty were female. All years of residency training were represented in the study. The third year residents represented the largest group of respondents (22.2%) and the fifth year residents the smallest (11.8%). The mean number of years since registration of the faculty was 12.3 years, with a standard deviation of 9.1 years. Table 1 shows participants' characteristics.

**Table 1:** Characteristics of residents and faculty who participated in SETQ.

	Residents	Faculty
Number invited	77	114
Number of respondents (response rate %)	66 (85.7)	99 (86.8)
Percentage respondents who are female	66.2	50.5
Mean age in years (standard deviation)	n/a*	50.8 (8.5)
Total number of residents' evaluations of faculty or faculty's self evaluations	613	99
Total number of residents who evaluated faculty and total number of faculty actually evaluated by residents (including faculty who did not self-evaluate)	66	114
Mean number of faculty evaluated by each resident (standard deviation)	9.3 (4.1)	n/a
Mean number of residents' evaluations per faculty member (standard deviation)	n/a	5.4 (2.6)
Percentage of residents per year of residency training		
First year	13.6	n/a
Second year	19.2	n/a
Third year	22.2	n/a
Fourth year	17.2	n/a
Fifth year	11.8	n/a
Sixth year	16.0	n/a
Mean number years of practice since first specialist registration as obstetrician and gynecologist (standard deviation)	n/a	12.3 (9.1)
Percentage of faculty who had formal training as clinical educators	n/a	69.7

n/a: not applicable.

*not inquired to assure residents' anonymity.

### Reliability and Validity

Factor loadings from exploratory factor analysis of residents' evaluations revealed a five composite scale structure. Due to low factor loadings, three items were eliminated after which factor analysis showed good stability. Each factor with its corresponding items and factor loadings is presented in table 2. Given the relatively small sample size for the faculty self-evaluations (99 records for structuring 23 items), it was not possible to conduct a stable factor analysis for the faculty instrument. Instead, we chose to apply residents' factor structure to faculty data to estimate the reliability of the five composite scales. Cronbach's alpha used as reliability coefficients were high for both residents' and faculty's composite scales, ranging from 0.84 to 0.94 among residents and from 0.76 to 0.89 among faculty. Item-total correlations yielded homogeneity within each composite scale.

**Table 2:** Characteristics of composite scales and items, with internal consistency reliability coefficient and corrected item-total correlations.

Item number	Scale and items†	Factor loadings on primary scale	Internal consistency reliability coefficient: Cronbach's α*		Corrected item-total correlations‡	
		Residents' evaluations	Residents' evaluations	Faculty self-evaluation	Residents' evaluations	Faculty self-evaluation
	*Learning climate*		0.84	0.76		
Q01	Encourages residents to participate actively in discussions	0.604			0.633	0.624
Q02	Stimulates residents to bring up problems	0.605			0.652	0.538
Q03	Motivates residents to study further	0.781			0.702	0.609
Q04	Stimulates residents to keep up with the literature	0.798			0.702	0.528
Q05	Prepares well for teaching presentations and talks	0.519			0.530	0.373
	*Professional attitude and behavior towards residents*		0.89	0.81		
Q06	Listens attentively to residents	0.771			0.715	0.617
Q07	Is respectful towards residents	0.807			0.747	0.700
Q08	Is easily approachable during on-calls	0.848			0.756	0.624
Q09	Is easily approachable for consultation	0.834			0.796	0.542
	*Communication of goals*		0.94	0.89		
Q10	States learning goals clearly	0.807			0.830	0.674
Q11	States relevant goals	0.827			0.885	0.846
Q12	Prioritizes learning goals	0.821			0.878	0.811
Q13	Repeats stated learning goals periodically	0.820			0.850	0.710
	*Evaluation of residents*		0.87	0.79		
Q14	Evaluates residents' specialty knowledge regularly	0.511			0.727	0.515
Q15	Evaluates residents' analytical abilities regularly	0.516			0.729	0.376
Q16	Evaluates residents' application of knowledge to specific patients regularly	0.500			0.720	0.419
Q17	Evaluates residents' medical skills regularly	0.668			0.745	0.734
Q18	Evaluates residents' surgical skills regularly	0.626			0.666	0.694
Q19	Educates about surgical skills in the operation room	0.778			0.481	0.504
	*Feedback*		0.88	0.86		
Q20	Gives positive feedback to residents regularly	0.597			0.641	0.673
Q21	Gives corrective feedback to residents	0.853			0.709	0.634
Q22	Explains why residents are incorrect	0.799			0.810	0.776
Q23	Offers suggestions for improvement	0.790			0.783	0.735

*Cronbach's α>0.70 was taken as an indication of satisfactory reliability of each composite scale.

† The items shared the same subject with different foregoing sentences “During my residency in obstetrics and gynecology, the attending faculty generally…” (residents' evaluation of faculty) or “In my role as an attending faculty, I generally…” (faculty self-evaluation).

‡ Item-total correlation values <0.3 indicate that the corresponding item does not correlate well with the composite scale.

As shown in table 3, inter-scale correlations were positive (P<0.01), implicating individual discriminating power of the five composite scales for both instruments. Correlation coefficients of the five composite scales and two global ratings ranged from 0.32 to 0.63 (P<0.01). As expected, each composite scale was moderately correlated with each of the two global ratings. Correlations are presented separately for residents and faculty in table 4.

**Table 3:** Inter-scale correlations† for residents' and faculty evaluations separately.

*Residents' evaluation of faculty*					
	Learning climate	Professional attitude and behavior towards residents	Communication of goals	Evaluation of residents	Feedback
Learning climate	1	0.405**	0.633**	0.551**	0.511**
Professional attitude and behavior towards residents		1	0.392**	0.331**	0.504**
Communication of goals			1	0.495**	0.499**
Evaluation of residents				1	0.504**
Feedback					1

*Faculty self-evaluation*					
Learning climate	1	0.428**	0.605**	0.549**	0.566**
Professional attitude and behavior towards residents		1	0.463**	0.436**	0.586**
Communication of goals			1	0.524**	0.461**
Evaluation of residents				1	0.569**
Feedback					1

*P<0.05,

**P<0.01.

† inter-scale correlations of <0.70 were taken as satisfactory indication of non-redundancy of each scale.

**Table 4:** Correlations† between scales and global ratings of (i) faculty being seen as an obstetric and gynecologic specialist role model and (ii) faculty's overall teaching qualities, estimated separately for residents' and faculty's evaluations.

	Faculty seen as an obstetric and gynecologic specialist role model		Faculty's overall teaching qualities	
	Residents' evaluations	Faculty self-evaluation	Residents' evaluations	Faculty self-evaluation
Learning climate	0.566**	0.464**	0.630**	0.620**
Professional attitude and behavior towards residents	0.632**	0.471**	0.538**	0.380**
Communication of goals	0.464**	0.455**	0.598**	0.603**
Evaluation of residents	0.434**	0.322**	0.494**	0.368**
Feedback	0.497**	0.561**	0.572**	0.500**

*P<0.05,

**P<0.01.

† correlation coefficients of 0.40–0.80 indicate valid measurements of faculty's teaching qualities by the SETQ instruments.

### Number of Residents' Evaluations Needed per Faculty

For a reliable evaluation of faculty's teaching qualities at least four residents' evaluations are needed per faculty. On average, there were 5.4 evaluations per faculty (standard deviation 2.6) with associated reliability coefficients ranging from 0.76 to 0.94 across scales and instruments. Calculations of the number of evaluations needed per faculty for different reliability coefficients showed that four to six evaluations per faculty would be needed at reliability coefficients no larger than 0.80 (table 5). Also, re-estimates of the reliability coefficients using sample data on faculty who were rated by 6 or less residents yielded reliabilities of >0.80.

**Table 5:** Number of residents' evaluations needed per faculty for reliable evaluation of faculty's teaching qualities for different reliability coefficients.

	Reliability coefficient of 0.60	Reliability coefficient of 0.70	Reliability coefficient of 0.80
Learning climate	4, 2	4, 3	5, 4
Professional attitude and behavior towards residents	4, 1	4, 2	5, 3
Communication of goals	4, 2	4, 4	5, 6
Evaluation of residents	4, 2	4, 3	5, 5
Feedback	4, 3	4, 5	5, 9

Numbers refer respectively to (i) extrapolation based on the simple formula in the methods section, (ii) the number of residents' evaluations needed per faculty based on interclass correlation type of reliability coefficients from multilevel models.

## Discussion

### Principal findings

This multicenter study found five important aspects of teaching with high reliability underlying the SETQ instruments. The high response rates and low number of evaluations needed for reliable assessment indicate the feasibility of the instruments for the evaluation of teaching qualities of individual obstetrics and gynecology faculty.

### Strengths and Limitations

One of the strengths of the SETQ instruments is the minimum of four evaluations needed to attain a reliable assessment of faculty's teaching qualities. This finding is congruent with the number of evaluations needed in the SETQ measurement instruments for anesthesiology faculty [16]. Other studies report seven to ten required evaluations [12], [28], [30]. The minimum of four evaluations decreases the workload on residents. Equal contributions of residents from all residency training years demonstrate a wide-ranging basis of participants.

The dependent relationship of residents towards faculty could present a potential difficulty. Residents might fear repercussions after giving negative feedback, especially in smaller departments. In an attempt to prevent this, the issue was discussed during the introduction of SETQ. Residents' anonymity was assured by returning the results on group level only and without mentioning sex or year of residency. High response rates from residents indicate an effective approach.

### Explanation and Interpretation

Clinical teaching improves when clinical educators receive feedback from their residents [11]. The SETQ system facilitates the provision of such feedback. Our study presents empirical support for the feasibility and psychometric qualities of the SETQ instruments for obstetrics and gynecology faculty.

The five composite scales from factor analysis of residents' evaluations correspond with factors discovered in previous research, adding to the internal consistency of the SETQ instruments [16], [18], [21]. Factor analysis of self-evaluation of anesthesiology faculty from one anesthesiology department resulted in five composite scales in spite of the smaller number of 36 participating faculty compared to the present study [16]. Uncovering composite scales within a homogeneous group (one residency training program) might require fewer evaluations as compared to a heterogeneous group of clinical teachers (nine residency training programs). Possibly, obstetrics and gynecology faculty from nine different training programs participating in this study do not share the same concept of teaching. This supports the need to investigate specialty-specific SETQ instruments.

Item-total correlation and inter-scale correlation were all within predefined limits, clearly adding to the validity of both obstetrics and gynecology instruments. Correlations between scales and the global rating of faculty's overall teaching qualities were higher compared to the global rating of faculty seen as an obstetrics and gynecologic role model (as expected), except for the composite scale ‘professional attitude and behavior towards residents’. Professional attitude and behavior towards residents is correlated more to being seen as an obstetrics and gynecologic role model compared to overall teaching qualities. Role modeling plays an important part in medical education, with great implications to improve teaching quality [31]. Another SETQ study investigated the association between teaching qualities of faculty and being seen as a specialist role model [32]. For obstetrics and gynecology, the professional attitude and behavior towards residents was the dominant predictor for faculty to be seen as an obstetrics and gynecology role model [32]. This offers support for specialty-specific analysis of SETQ instruments, as other specialties showed different dominant predictors such as feedback or learning climate.

### Implications for Clinical Education, Research and Policy

Teaching and role modeling can be learned and it is helpful to receive feedback to define one's individual developmental trajectory [4], [33], [34]. The SETQ system enables faculty to evaluate their performance in subsequent years. Continuous measurements provide follow-up information for lifelong learning of professionals. Faculty should preferably take an active approach in lifelong learning and identifying learning needs is a crucial first step in this process [35]. More research is needed to develop reliable benchmarks and analyze the use of narrative feedback. The differences between outcomes from successive evaluations can provide insight in the effect of SETQ [11], [27]. Future research should focus on the effectiveness of SETQ in improving teaching quality as perceived by residents and faculty. Over time, the SETQ study aims to investigate the effect of the quality of teaching on the quality of care.

### Conclusions

This study supports the reliability and validity of both resident – and faculty completed instruments underlying the SETQ system for obstetrics and gynecology faculty. Implementation seems attainable in both academic and non-academic training programs. Reliable individual feedback reports can be generated based on a minimum of four evaluations. Faculty may use their individual feedback reports for reflection and designing personal development tracks. The combination of the two instruments in the SETQ system offers a valuable structure to evaluate teaching qualities of obstetrics and gynecology faculty. For faculty it means they are provided with the possibility to improve their teaching in order to facilitate high quality of future doctors.
